# A “Prime-Pull” Vaccine Strategy Has a Modest Effect on Local and Systemic Antibody Responses to HIV gp140 in Mice

**DOI:** 10.1371/journal.pone.0080559

**Published:** 2013-11-19

**Authors:** John S. Tregoning, Viviana Buffa, Anna Oszmiana, Katja Klein, Adam A. Walters, Robin J. Shattock

**Affiliations:** Mucosal Infection & Immunity Group, Section of Infectious Diseases, Imperial College London, London, United Kingdom; Commissariat a l'Energie Atomique(cea), France

## Abstract

One potential strategy for the prevention of HIV infection is to induce virus specific mucosal antibody that can act as an immune barrier to prevent transmission. The mucosal application of chemokines after immunisation, termed “prime-pull”, has been shown to recruit T cells to mucosal sites. We wished to determine whether this strategy could be used to increase B cells and antibody in the vaginal mucosa following immunisation with an HIV antigen. BALB/c mice were immunised intranasally with trimeric gp140 prior to vaginal application of the chemokine CCL28 or the synthetic TLR4 ligand MPLA, without antigen six days later. There was no increase in vaginal IgA, IgG or B cells following the application of CCL28, however vaginal application of MPLA led to a significant boost in antigen specific vaginal IgA. Follow up studies to investigate the effect of the timing of the “pull” stimulation demonstrated that when given 14 days after the initial immunisation MPLA significantly increased systemic antibody responses. We speculate that this may be due to residual inflammation prior to re-immunisation. Overall we conclude that in contrast to the previously observed effect on T cells, the use of “prime-pull” has only a modest effect on B cells and antibody.

## Introduction

One strategy for HIV vaccine development is to generate a local immune barrier at the site of infection [1]. Evidence demonstrating that in the majority of heterosexual transmission cases, infection is caused by a single founder virion [2] suggests that this strategy could be effective. Whilst mucosal lymphoid cells – including T cells, intra-epithelial lymphocytes and innate lymphoid cells can play a role in local protection, antibody is a potent tool to provide the local immune barrier [3]. The ideal result of HIV vaccination would be the generation of broadly neutralising antibodies at the site of infection [4], but virus specific IgA could play a role in the immune barrier due to its immune exclusion function, even if it is not directly neutralising [5].

We have previously observed that mucosal immunisation can induce local antibody responses to trimeric HIV envelope protein gp140 [6]–[8]. One possible approach to increase mucosal responses is to use a “prime-pull” strategy, where lymphocytes are redirected to local sites using chemokines following immunisation. This strategy has been demonstrated to be effective for the recruitment of both CD4 and CD8 cells to the vagina using CCL9 and CCL10 [9] and regulatory CD4 T cells to the lungs using CCL17 and CCL22 [10]. We wished to determine whether a similar approach could be used to recruit B cells to the vagina following immunisation.

B cells are attracted to a range of factors, including the chemokines CCL19, CCL21, CCL28, CCL25, the integrins α_4_β_1,_ and α_4_β_7_ and the cytokines BAFF, APRIL and TSLP [11]. We have previously looked at the effect of BAFF, APRIL and TSLP as mucosal adjuvants [12] and observed that only TSLP boosted the antibody response to antigen. The chemokine receptors CCR7 and to some extent CXCR4, are required for naïve B cell entry into lymph nodes and migration to the T cell zones [13], and antigen exposure increases CCR7 expression and the chemokine CCL19 is effective when used as an adjuvant [14]. But we are aiming to recruit plasmablasts and/or plasma cells – which are CCR7 negative. The chemokine CCL28 attracts B cells to the mucosa, particularly IgA producing cells [15]. CCL28 is expressed by mucosal epithelia at the bronchi, salivary gland, mammary glands and small intestine and when co-administered with HIV-VLP, CCL28 boosted the antibody response [16]. One limitation of translating the chemokine strategy to a vaccine is that because chemokines are proteins, they are expensive to manufacture, therefore we wished to determine whether Toll like receptor (TLR) ligands which have been used as mucosal adjuvants [17] can be used in the “prime-pull” approach. One such agent is monophosphoryl lipid A (MPLA) a non-toxic derivative of LPS, the first TLR ligand approved for human use for its safety and effectiveness as an adjuvant [18].

In this study we investigated the use of the chemokine CCL28 and TLR ligand MPLA as boost agents (without antigen) in a “prime-pull” regime following either mucosal or systemic immunisation with the HIV envelope protein gp140. We observed that the vaginal administration of MPLA alone after immunisation but not CCL28 led to an increase in vaginal IgA, systemic IgA and IgG and antigen specific B cells in the female genital tract. The timing of boost was important, with a greater response seen when “pull” stimulation was given 7 or 14 days after immunisation compared to when it is given on the day of immunisation. Interestingly mucosal administration of MPLA alone significantly increased systemic antibody responses to subsequent immunisations. Here we show that it is possible to increase the vaginal IgA using a “prime-pull” strategy, but the increase in antibody titre was modest and unsustained.

## Materials and Methods

### Animals, Antigen and adjuvants

Female BALB/c mice, 6–8 week old, were obtained from Harlan Olac Ltd (Bevil's Hill, UK). All procedures were performed in accordance with the United Kingdom's Home Office standards under the Animals Scientific Procedures Act, 1986, and approved by the Ethical Review Boards at Imperial College London and at St George's University of London. In the timecourse and comparison of TLR ligands studies, the same group of control animals were used to reduce animal usage. A clade C HIV-1 envelope clone p97CN54 was originally isolated from a Chinese patient [19] and was made available by H. Wolf and R. Wagner, University of Regensburg, Germany. Trimeric gp140 (gp120 plus the external domain (ED) of gp140), designated CN54 gp140, was produced as a recombinant product in CHO cells and manufactured to GMP specification by Polymun Scientific, Vienna, Austria. The TLR ligand FSL-1 (TLR2/6) was purchased from Invivogen, monophosphoryl Lipid A (MPLA, TLR4) from Sigma and CpG (TLR9) from MWG. Recombinant murine CCL28 was purchased from R&D systems.

### Immunisation protocol

Mice were immunized 3 times with 3 weeks interval, with 10 µg gp140 and 10µg MPLA. The gp140/MPLA formulation was either administered intranasally in a total volume of 20µl or subcutaneously in a volume of 50µl. At 0, 7, or 14 days after immunisation, animals received an intravaginal “pull” stimulation of 10µg of MPLA, CCL28, FSL-1 or CpG in a volume of 20µl.

### Sample and tissue collection

Serum and mucosal samples were obtained at various intervals before or after immunisation as described previously [12].

The mouse genital tract, including vagina, uterus, oviducts, and ovaries were dissected from the animal, and placed in cold complete medium (RPMI-1640 supplemented with 10% fetal bovine serum (FBS), 2 mM glutamine, 10 mM HEPES, 100 IU/ml penicillin, 100µg/ml streptomycin, and 10µg/ml gentamycin). The tissue was finely cut with a scalpel, washed with complete medium and digested at 37°C for 1 h on a shaker with 5 ml of serum-free RPMI-1640 medium that contained 2 mg Collagenase Dispase and 0.1 mg/ml DNaseI (Roche Diagnostics). The digested tissue was spun and the cell pellet washed twice in CM. The lymphoid cell population was separated from the stromal cells by density gradient centrifugation (Lympholyte, Cedarlane Laboratories).

### Detection of antigen-specific antibody responses by ELISA

MaxiSorp 96-well plates were coated overnight with 1.0 µg/ml HIV-1 gp140 in PBS. Plates were blocked for 1 h at 37°C with 1% BSA in PBS. Serially diluted samples were incubated for 1 h at 37°C. Bound IgG was detected by incubation for 1 h at 37°C with horseradish peroxidase (HRP)-conjugated goat anti-mouse IgG (AbD Serotec, UK) and IgA was detected with biotin-conjugated goat anti-mouse IgA (AbD Serotec, UK). For IgA detection, plates were incubated with Streptavidin-HRP (R&D Systems) for 1 h at 37°C. Plates were developed using tetramethylbenzidine (TMB) substrate. The reaction was stopped with stop solution (1N H_2_SO_4_) and read at 450 nm. Reciprocal endpoint titres were calculated by using GraphPad Prism 4 using a cut-off value at OD_450_ of 0.1 for all samples.

### ELISPOT

Cells from genital tract were assessed for the presence of gp140-specific IgG and IgA antibody secreting cells (ASC). Cells from genital tract were assessed immediately after isolation. ELISPOT assays were performed using a commercial kit from MABTECH (Nacka Strand, Sweden) following the manufacturer's recommendations. The spots were counted using the AID ELISPOT reader ELR03 (Autoimmune Diagnostika).

### Statistical Analysis

Analyses were performed using GraphPad Prism, version 4.00 (GraphPad Software). Statistical differences between groups were calculated using one-way analysis of variance (ANOVA) with appropriate post tests to measure significance between pairs of groups.

## Results

**Figure 1:**
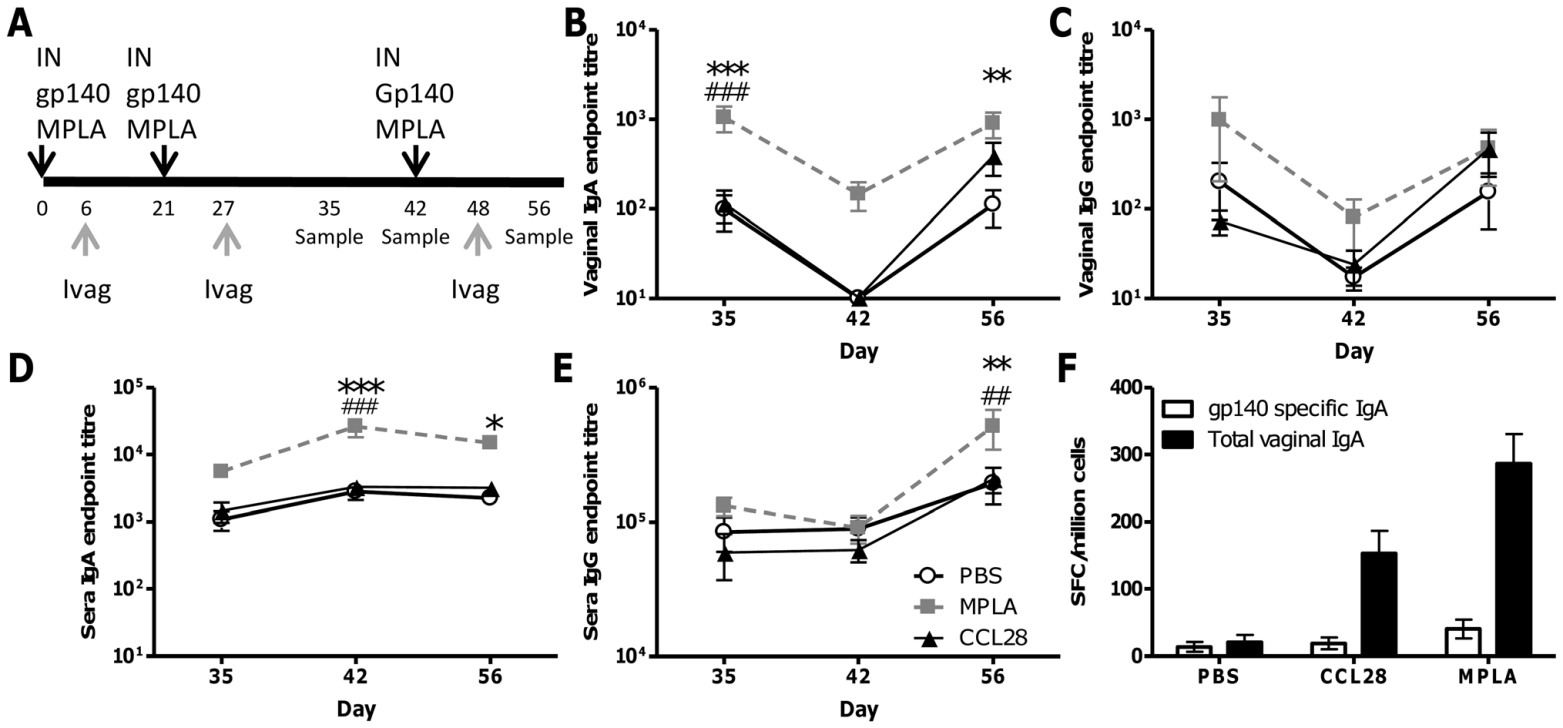
Intravaginal MPLA can boost local IgA responses after mucosal immunisation. BALB/c mice were immunised intranasally (IN) with 10µg gp140+MPLA, 6 days later they received 10µg of CCL28 or MPLA or PBS control without antigen intravaginally (Ivag) (A). Gp140 specific IgA and IgG were measured in vaginal lavage (B, C) or sera (D,E) at various timepoints after immunisation. Anti-gp140 and total IgA ASC were measured by ELISPOT in pooled female genital tracts of mice at day 56 (F). Data points represent mean +/− SEM of n = 8 animals from 1 experiment except panel F where the bar represents mean of n = 3 pooled samples, *p<0.05, ** p<0.01, *** p<0.001 comparing MPLA and PBS groups, # p<0.05, ## p<0.01, ### p<0.001 comparing MPLA and CCL28 groups.

We wished to determine whether we could recruit B cells into the vaginal mucosa following immunisation using a “prime-pull” strategy. Mice were immunised three times intranasally with the model HIV antigen gp140 together with MPLA as a mucosal adjuvant, which we have previously shown to increase systemic and local responses to antigen [17]. Six days after each immunisation or “prime”, mice received a vaginal administration of 10µg MPLA, 10µg CCL28 or PBS control, without antigen designed to provide a chemotactic “pull” to coincide with release of antigen specific plasmablasts into the systemic circulation. Thus each animal received three rounds of “prime” immunisation followed by vaginal “pull” stimulation (Fig 1A). Anti-gp140 IgG and IgA were measured in sera and vaginal washes collected on days 34 – after 2^nd^ intravaginal “pull” dose, 42 – before the 3^rd^ intravaginal “pull” dose and 56 after the 3^rd^ intravaginal dose. “Pull” stimulation with CCL28 had no significant effect on antibody specific IgG or IgA in serum or vaginal lavage compared to PBS treated animals. However, mice that received intravaginal MPLA had significantly greater levels of vaginal IgA levels after “pull” stimulation on day 34 (Fig 1B, p<0.01). Although these responses appeared to wane after the second administration of MPLA, levels rose again after the third intravaginal dose (day 56, p<0.01). There was no significant difference in mucosal IgG after MPLA delivery (Fig 1C). The MPLA treated group also had significantly more sera IgA (Fig 1D, p<0.05) on days 42 and 56 and sera IgG on day 56 compared to the PBS treated animals (Fig 1E, p<0.01). Total and antigen specific B cells were measured in the female genital tract by ELISPOT, due to the low number of cells collected, samples had to be pooled for analysis. A trend of increased B cell numbers was observed in the vaginas of MPLA treated mice – both total and antigen specific (Fig 1F).

**Figure 2:**
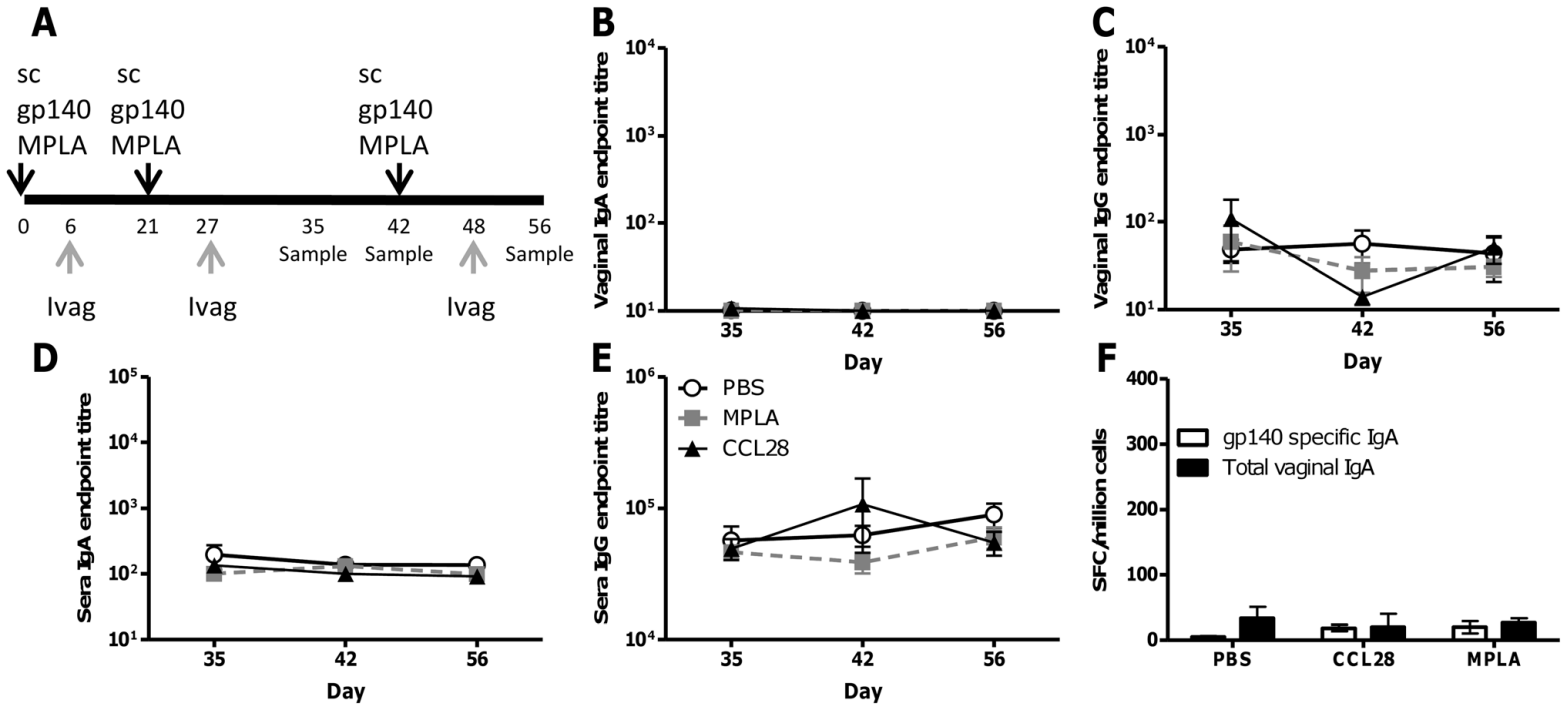
Intravaginal MPLA does not boost local IgA responses after systemic immunisation. BALB/c mice were immunised subcutaneously (sc) with 10µg gp140+MPLA, 6 days later they received 10µg of CCL28 or MPLA or PBS control without antigen intravaginally (A). Gp140 specific IgA and IgG were measured in vaginal lavage (B, C) or sera (D,E) at various timepoints after immunisation. Anti-gp140 and total IgA ASC were measured by ELISPOT in pooled female genital tracts of mice at day 56 (F). Data points represent mean +/− SEM of n = 8 animals from 1 experiment except panel F where the bar represents mean of n = 3 pooled samples.

Having observed an increase in vaginal responses following MPLA treatment of intranasally immunised mice, we wished to determine whether it was possible to “pull” cells into the mucosa following a systemic immunisation. This would be advantageous as the initial vaccination could be delivered using standard methodology, improving the practicality of the approach. As with the intranasal experiment, mice were immunised three times subcutaneously with gp140 and MPLA, followed by intravaginal administration of MPLA, CCL28 or PBS 6 days after each immunisation (Fig 2A). As observed previously [17], there was no detectable mucosal IgA (Fig 2B) but detectable levels of mucosal IgG (Fig 2C), sera IgA (Fig 2D) and IgG (Fig 2E) after subcutaneous immunisation. It is of note that the sera IgA was 2 logs lower than seen after intranasal immunisation. Intravaginal “pull” stimulation with either MPLA or CCL28 had no effect on levels of antigen specific antibody (IgA or IgG) in sera or mucosally or B cell recruitment to the female genital tract (Fig 2F). Comparing the subcutaneous immunised groups with the intranasally immunised groups, we observe that intranasal immunisation gave greater mucosal levels of antibody and sera IgA, but equivalent sera IgG to subcutaneous immunisation. Interestingly intravaginal MPLA significantly increased the level of sera IgG in the intranasal vaccine group but not the subcutaneous vaccine group (Fig 1E and Fig 2E).

**Figure 3:**
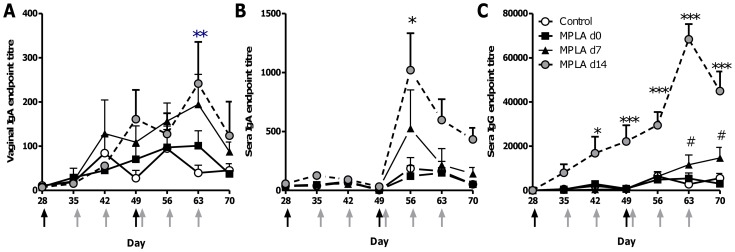
The timing of intravaginal MPLA application affects the boosting of local responses. BALB/c mice were immunised intranasally with 10µg gp140+MPLA (black arrows), they received 10µg of MPLA alone intravaginally on d0, d7, or d14 post intranasal immunisation (grey arrows). Gp140 specific IgA was measured in vaginal lavage (A) and gp140 specific IgA (B) or IgG (C) in sera at various timepoints after immunisation. Data points represent mean +/− SEM of n = 5 animals from 1 experiment, *p<0.05, **p<0.01, *** p<0.001 comparing MPLA d14 and control groups, # p<0.05 comparing MPLA d7 and control groups.

The observation that MPLA was effective at influencing vaginal IgA responses following intranasal rather than systemic immunisation fits with previous observations suggesting immunological linkage between the upper respiratory and lower genital tract of mice [20] and the preferential induction of IgA responses via mucosal immunisation. Subsequent experiments were performed to determine whether the timing of vaginal treatment with MPLA following intranasal immunisation altered the effect on local antibody responses. Peak plasmablast release into the circulation is thought to occur approximately 7 days after immunisation disappearing by day 14, while the accumulation of memory B cells in the circulation occurs 14–28 days after immunisation [21]. To determine the differential impact on these B cell populations, mice were immunised three times intranasally with a gp140 and MPLA “prime” and intravaginal MPLA “pull” on either 0, 7 or 14 days after immunisation. Antigen specific IgA and IgG were measured in sera and vaginally at seven day intervals. Intranasal immunisation alone led to transient antigen specific IgA and IgG in the sera after the third immunisation and IgA in the mucosal lavage, no antigen specific IgG was detectable in the vaginal lavage of any group at d56, 63 or 70. Administration of intravaginal MPLA on the day of immunisation had no effect on IgA levels in the vagina (Fig 3A) or the sera (Fig 3B) or serum IgG (Fig 3C) compared to the control. Vaginal specific IgA responses were significantly increased on day 63 after d14 “pull” stimulation (Fig 3A, p<0.05), declining by day 70. Administration of intravaginal MPLA on d7 after immunisation transiently enhanced serum specific IgA levels while administration on d14 significantly raised specific IgA in the sera on day 56 (Fig 3B, p<0.05). Intravaginal administration of MPLA on day 14 also led to a significant increase in antigen specific IgG in the sera after the final intranasal immunisation at days 56, 63 and 70 (Fig 3C, p<0.01), with d7 administration leading to a slight increase at d63 and d70.

**Figure 4:**
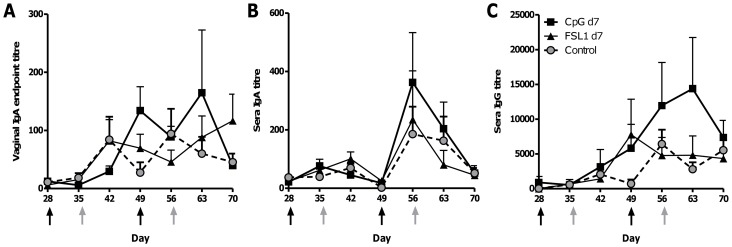
The selection of TLR ligand affects the boosting of local responses. BALB/c mice were immunised intranasally with 10µg gp140+MPLA (black arrows), they received 10µg of CpG or FSL-1 alone intravaginally on d7 post intranasal immunisation (grey arrows). Gp140 specific IgA was measured in vaginal lavage (A) and gp140 specific IgA (B) or IgG (C) in sera at various timepoints after immunisation. Data points represent mean +/− SEM of n = 5 animals from 1 experiment.

We have previously observed differences in the adjuvant effects of different TLR agonists applied mucosally in mice [17]. We wished to determine if there was a difference in effect with respect to intravaginal stimulation of local antibody response. Mice were immunised three times intranasally with the model HIV antigen gp140 plus MPLA as a mucosal adjuvant and received intravaginal dosing with 10µg of either CpG (TLR9 agonist) or FSL-1 (TLR2/6 agonist) 7 days after immunisation. CpG appeared to induce the highest level of specific IgA in the vagina, although due to high variability this did not reach statistical significance (Fig 4A). FSL-1 administration had no effect on local IgA responses. The third intranasal immunisation led to increased levels of sera IgA (Fig 4B) and IgG (Fig 4C), but there was no significant difference between the “pull” TLR ligand used.

## Discussion

In this study we wished to determine whether the local administration of a stimulatory agent was able to increase local antibody levels. We observed a modest, transitory increase in local IgA with the TLR4 ligand MPLA, but no effect with the chemokine CCL28. Boosting of local IgA only occurred when the mice were immunised intranasally and not when they were immunised systemically. The timing of boost was important, boosting on the day of immunisation had no effect on the local titre, but boosting on d7 or d14 after immunisation increased the local response and had a sustained effect on sera IgA and IgG levels.

In contrast to the recruitment of T cells to the vaginal mucosa with CCL9 and CCL10 after parenteral HSV immunisation [9], we saw no significant increase in local antibody responses when using the chemokine CCL28. One possibility for this is that CCL28 may have not been the most effective chemokine to use, whilst it has been shown to be important in homing to the mammary gland [22] and the gut [23], homing requirements for the vagina may be different. Other chemokines involved in the recruitment of plasma cells or plasma blasts might have been more effective. Possibilities include CXCL12 which engages CXCR4 and is involved in plasma cell retention in the bone marrow [24]; CXCL9, CXCL10 and CXCL11 which engage CXCR3 and lead to the recruitment of plasma cells to inflamed sites [25]; CCL25 which engages CCR9 and is involved in the recruitment of IgA plasma cells to the small intestine [23] and CCL19 which we have shown can be effective when used as an adjuvant [14]. Previous studies used a cocktail of chemokines and it may be that a single chemokine is insufficient to recruit cells and B cells may require additional signals for recruitment to mucosal sites than chemokine alone for example up-regulation of integrins including α_4_β_7_ [26], which have been shown to be more important in IgA cell recruitment to the gut than CCL28 or CCL25 [27]. It is possible that the dose of CCL28 used (10µg) was insufficient for the recruitment of B cells, but the dose used was greater than the dose of chemokine used in the HSV [9] or RSV [10] studies. Appropriate formulation might enhance localized delivery of CCL28, however greater doses of recombinant protein would significantly reduce the potential translation of such an approach to humans when accounting for differences in body mass and would have significant cost implications for any potential prime-pull vaccine strategy.

We did however see some effect of intravaginal boosting with MPLA at day seven after immunisation. We hypothesize that the mechanism by which this works is that the TLR ligands induce local inflammation which leads to cellular recruitment to the vagina. This is supported by the ELISPOT data which showed that MPLA delivery increased numbers of both specific and non specific IgA producing cells in the vagina. Previously it has been shown that mice expressing a constitutively active form of TLR4 (the receptor for MPLA) express higher levels of CCL20, CCL28 and APRIL in intestinal epithelium [28]. Speculatively, this may explain why TLR ligands were more effective than the application of a single chemokine alone as they could induce a mixture of factors. It was of interest that MPLA administration after intranasal, but not subcutaneous immunisation significantly increased serum antibody responses at the final immunisation. This effect may be caused by residual inflammation caused by the MPLA prior to subsequent immunisation. We hypothesize that the MPLA is acting directly on B cells, priming them for antigen re-exposure rather than acting on antigen presenting cells for two reasons. Firstly the gap in timing between the MPLA administration and subsequent immunisation is seven days, during which time it would be anticipated that the inflammation would have resolved. Secondly, the MPLA administration is at a distal site to the immunisation, so any activated antigen presenting cells would need to be circulating to the immunisation site. The context of immunisation is important and this data suggests that previous TLR stimulation may alter the outcome of subsequent immunisation. It was of note that MPLA administration only had an effect on mucosally primed animals and not systemically primed animals, suggesting that the context of vaccination is important in B cell homing and circulation [12], [17].

There are a number of caveats that may influence translation of this approach to human vaccines. The first limitation is the modest effect we observed in this study, but there may be differences going into humans, especially due to the differences in TLR responses between mice and humans. The second limitation is of a practical nature – particularly as HIV vaccines are most relevant in resource poor settings. Requiring individuals to return for a second visit seven days after each immunisation would be extremely restrictive, this could potentially be circumvented with home administration of the boost – if formulated in a user friendly form. But, there may be issues with the cultural acceptability of the vagina for drug delivery. There is also a chance that inducing immune activation in the vagina could increase the risk of transmission [29]. Another issue is that this protection would only be effective in controlling male to female vaginal transmission, though it may be possible to boost immune responses with penile or rectal boosting. In conclusion whilst we observed a modest increase of local and systemic antibody responses when MPLA was used as a local boost we do not believe that this approach is appropriate for future vaccine development.
